# 索拉非尼联合维奈克拉、阿扎胞苷方案诱导治疗FLT3-ITD突变阳性急性髓系白血病12例的疗效及安全性分析

**DOI:** 10.3760/cma.j.issn.0253-2727.2022.11.013

**Published:** 2022-11

**Authors:** 昊 艾, 瑞华 米, 琳 陈, 旭 纪, 青松 尹, 旭东 魏, 永平 宋

**Affiliations:** 郑州大学附属肿瘤医院血液科、河南省肿瘤医院血液科，郑州 450008 Department of Hematology, the Affiliated Cancer Hospital of Zhengzhou University, Zhengzhou 450008, China

目前，国内外报道索拉非尼单药及联合化疗治疗FLT3-ITD突变阳性急性髓系白血病（AML）患者均有效[1]–[2]，但对于老年、机体状态差、合并脏器功能减退、重症感染及复发后诱导治疗不易缓解患者，单药及联合高强度化疗难以耐受且缓解率低。因此，如何选择与索拉非尼协同，既能提高缓解率又能减少相关不良反应的方案尤为重要。近年来，国内外研究及临床实践均表明维奈克拉（VEN）联合阿扎胞苷（AZA）已成为老年、不适合高强度化疗初治及复发患者的新选择[3]。我们尝试采用索拉非尼联合VEN+AZA方案诱导治疗FLT3-ITD突变阳性AML，并对其临床疗效及不良反应进行初步观察。

## 病例与方法

1. 病例：回顾性分析2019年12月至2021年12月郑州大学附属肿瘤医院收治的12例应用索拉非尼联合VEN、AZA方案治疗的FLT3-ITD突变阳性AML患者，所有患者均经MICM（细胞形态学、免疫学、细胞遗传学、分子生物学）分型检查确诊，诊断和分型符合文献[4]标准。12例患者中男4例，女8例，中位年龄57.5（21～78）岁。8例为初治患者，4例为复发难治患者。FAB分型：M_5_ 9例，M_4_ 3例。8例患者治疗前存在感染、心功能不全等并发症。治疗前中位骨髓幼稚细胞比例为54.4％（15.0％～91.4％）。所有患者均伴有FLT3-ITD基因突变，同时合并NPM1突变4例，DNMT3A突变2例，ACBC1突变2例，RUNX1突变2例，TET-2、IDH1、KRARS1、SRSF2突变各1例，12例患者的临床资料详见表1。

**表1:** 应用索拉非尼联合VEN、AZA方案治疗的12例FLT3-ITD突变阳性急性髓系白血病患者的临床情况、疗效及转归

例号	性别	年龄(岁)	FAB分型	FLT3-ITD突变率(%)	其他突变	染色体核型	合并症	疾病状态	疗效	转归
1	女	68	M_5_	20.8	IDH1	46,XX,del(5)(q13;q33)[10]	肺部感染	初治	CR	巩固7个周期复发，苯磺酸克立福替尼再诱导，持续CR 8个月余
2	男	73	M_5_	56.8		正常核型	肺部感染肠道感染	初治	CR	因上呼吸道出血延迟治疗，MRD增高，索拉非尼联合VEN+AZA+HHT、VEN+地西他滨巩固2个周期后复发。持续CR 5个月余
3	女	69	M_4_	19.6	NPM1、WT1 RUNX1	正常核型		初治	CR	巩固治疗中，持续CR 8个月余
4	女	63	M_4_	74.0		正常核型		初治	CR	巩固治疗中，持续CR 8个月余
5	女	66	M_5_	47.0	DNMT3A、NPM1	正常核型	心功能不全	初治	CR	巩固治疗1个周期后失访，持续CR 2个月余
6	男	70	M_4_	21.0	DNMT3A、NPM1A、CBC1、ABL1	正常核型	肺部感染心功能不全	初治	CR	巩固治疗9个周期后，索拉非尼联合干扰素+白细胞介素2维持治疗，持续CR 15个月余
7	男	36	M_5_	57.4	KRARS	46，XY，t(3;21)(q24;q22)[10]	肺部感染心功能不全肠道感染	初治	CRi	持续CRi 2个月余失访
8	男	78	M_5_	16.0	SRSF2、RUNX1	47,XY+13[8]/46,XY[2]	肺部感染心功能不全	初治	PR	应用SKLB1028胶囊PD,予索拉非尼联合VEN+AZA+HHT达CR 1个月余
9	女	21	M_5_	54.2	FAT1、ABCB1	正常核型	肺部感染	IA、CLAG方案达PR	CR	行亲缘单倍体造血干细胞移植，持续CR 21个月余
10	女	60	M_5_	35.5		正常核型		IA、Ara-C、DCHAG、干白沙维持治疗后复发	CR	巩固治疗2个周期后失访，持续CR3个月余
11	女	37	M_5_	19.7	NPM1	正常核型	肺部感染	IA方案达PR	CRi	巩固治疗1个周期后再次复发，行亲缘单倍体造血干细胞移植后出现aGVHD及肺部感染，放弃治疗
12	女	50	M_5_	30.0	TET-2	47,XX,+8[5]/46,XX[4]	肺部感染	IA、HEA、CLAG、地西他滨+CAG达PR	PR	诱导治疗达PR 1个月后失访

注：VEN：维奈克拉；AZA：阿扎胞苷；HHT：高三尖杉酯碱；IA：盐酸伊达比星+阿糖胞苷；Ara-C：阿糖胞苷；HEA：高三尖杉酯碱+阿糖胞苷+依托泊苷；CLAG：克拉屈滨+阿糖胞苷+G-CSF；CAG：盐酸阿柔比星+阿糖胞苷+G-CSF；DCHAG：地西他滨+CAG+高三尖杉酯碱；干沙白：干扰素+沙利度胺+白细胞介素2；MRD：微小残留病；CR：完全缓解；CRi：完全缓解伴血细胞不完全恢复；PR：部分缓解；PD：疾病进展；aGVHD：急性移植物抗宿主病

2. 治疗方案：索拉非尼联合VEN+AZA方案：索拉非尼每次400 mg，口服，每日2次；VEN 100 mg第1天，200 mg第2天，300 mg第3天，400 mg第4～28天，口服，每日1次（索拉非尼及VEN用量根据用药期间耐受性、合并用药及骨髓抑制情况调整）；AZA 75 mg/m^2^，每日1次，第1～7天。采用伏立康唑及泊沙康唑预防及治疗真菌感染时，调整VEN至200 mg，口服，每日1次。若出现Ⅳ度骨髓抑制，同时合并重症感染时，可停用VEN。治疗第14天复查骨髓情况，若原始细胞<5％，继续予以口服VEN；若下降比例>50％但未达<5％，继续口服VEN同时有检测条件的单位应检测VEN血药浓度；若无效，更改方案再诱导治疗。达完全缓解（CR）后行巩固治疗，有合适供者且符合要求者行异基因造血干细胞移植（allo-HSCT）。

3. 治疗期间不良事件的发生及处理原则：按照WHO急性和亚急性不良反应分级标准评定不良反应。治疗期间定期监测血常规和肝肾功能，针对药物引起的骨髓抑制，采取输注相应血制品及使用促造血生成药物。当患者出现发热或寒战合并感染时，常规进行血培养及病毒检测，并按经验予以广谱抗生素治疗，同时予以口服伏立康唑（200 mg，每日2次）或泊沙康唑（5 ml/次，每日4次，间隔6 h）预防真菌感染，常规抗生素治疗5～7 d无效或者CT提示真菌感染者给予静脉抗真菌治疗。

4. 疗效及药物不良反应评价标准：所有患者治疗前完成下列检查：血细胞计数及分类，心、肝、肾功能检查，骨髓细胞形态学，流式细胞术免疫表型，染色体核型分析及基因突变。疗效判定依据国际工作组（IWG）提出的疗效标准。在1个疗程治疗结束，血常规指标回升后再次行骨髓穿刺术评估疗效。疗效分为CR、CR伴血细胞不完全恢复（CRi）、部分缓解（PR）、疾病进展（PD），达到CR、CRi、PR为治疗有效。不良反应分级依据美国国立癌症研究所常见毒性判定标准（第2版）进行判定。

5. 随访：随访截至2022年3月10日。中位随访时间200（30～630）d。末次随访疾病状态包括持续缓解、复发、失访。随访方式包括电话随访、门诊复查、查阅病历。

6. 统计学处理：采用SPSS19.0软件进行统计学分析。治疗前后骨髓幼稚细胞比例比较采用配对*t*检验，*P*<0.05为差异有统计学意义。

## 结果

1. 疗效评价：所有患者均顺利完成诱导治疗。12例患者接受索拉非尼联合VEN+AZA方案治疗后总有效率为100％。治疗后骨髓幼稚细胞比例明显下降［3.9％（0.5％～24.0％）对54.4％（15.0％～91.4％），*P*<0.01］。7例达到CR，3例达CRi，2例达PR，达CR中位时间32（22～53）d。达分子生物学缓解（FLT3-ITD突变转阴）中位时间为60（44～76）d。其中初治患者8例，3例达CR，4例达CRi，1例达PR；4例复发难治患者中2例达CR，1例达CRi，1例达PR，其中2例复发患者序贯亲缘单倍体造血干细胞移植。上述患者中位无复发生存时间为6（1～21）个月。具体疗效及转归见表1。

2. 不良反应：治疗过程中所有患者均出现Ⅲ～Ⅳ血液学不良反应，粒细胞缺乏中位时间18（15～40）d，中位HGB最低值55（50～70）g/L，中位PLT最低值16（6～25）×10^9^/L，予以G-CSF及输血对症支持治疗后好转。非血液学不良反应主要为粒细胞缺乏期感染，其中合并肺部感染3例，肠道感染2例，予以抗细菌联合抗真菌治疗好转。6例患者发生Ⅰ～Ⅱ级恶心、呕吐，予以止吐等对症治疗后均好转。未见Ⅲ～Ⅳ级非血液学不良反应，所有患者未出现肿瘤溶解综合征。

## 讨论

FLT3突变在正常核型AML中发生率为28％～33％[5]，多项回顾性研究结果显示，FLT3-ITD突变与高原始细胞、低缓解率、低无病生存率及低总生存率相关，NCCN指南将其作为独立于染色体核型的预后不良因素[6]。随着索拉非尼、米哚妥林、奎扎替尼、吉瑞替尼等一系列FLT3-ITD抑制剂应用于临床，使得此类AML疗效得到改善[7]–[8]，但由于价格等因素，目前国内临床主要以索拉非尼为主。

索拉非尼作为以FLT3为靶点的小分子酪氨酸激酶抑制剂，通过与酪氨酸激酶竞争ATP结合位点而抑制其活性，干扰信号转导，促使AML细胞分化、凋亡，体外实验表明索拉非尼可明显抑制FLT3-ITD、TKD突变AML细胞株增殖，促使分化并诱导凋亡[9]。国内外研究表明索拉非尼单药及联合化疗可有效降低外周血及骨髓原始幼稚细胞，但索拉非尼单药治疗总反应率及CR率均较低[1]，[10]。因此，为提高FLT3-ITD突变阳性AML疗效，国内外不同中心进行了索拉非尼联合IA（去甲氧柔红霉素+阿糖胞苷）方案、DA（柔红霉素+阿糖胞苷）方案及CAG、CHAG预激方案的尝试，使得患者的CR率得到明显提高，但老年、合并感染、化疗耐受性差及复发患者疗效欠佳[2]，[11]。因此，对于发病初期年龄大、机体状态差、合并脏器功能减退、重症感染及存在不良遗传学改变、复发FLT3-ITD突变阳性AML如何选择合适方案配伍索拉非尼，从而在减少相关不良反应的同时提高疗效值得探讨。

B细胞淋巴瘤2（Bcl-2）蛋白家族在AL、慢性淋巴细胞白血病（CLL）及淋巴瘤等血液系统恶性肿瘤中高表达，其参与调控线粒体细胞凋亡过程，促使凋亡蛋白进入线粒体，激活caspase级联反应，从而促使细胞凋亡[12]。Bcl-2抑制剂VEN作为唯BH3蛋白类似物，通过与Bcl-2蛋白结合，替换Bim等凋亡蛋白，导致线粒体外膜通透性改变、半胱氨酸蛋白酶激活，促进细胞凋亡。此外，VEN虽然减少Bim与Bcl-2的结合，但却增加了Bim与Mcl-1的结合，从而阻止AML细胞凋亡，这也是Bcl-2抑制剂耐药的可能机制之一[13]。因此，多项临床研究表明，VEN单药治疗复发及不能耐受强化疗AML虽然有效，但整体CR+CRi率仅为20％左右，且维持有效时间短，而AZA则通过降低Mcl-1水平，协同VEN激活线粒体细胞凋亡，提高抗白血病效应[14]。DiNardo等[15]将431例初治老年AML患者随机分为VEN联合AZA组及对照组，CR率分别为36.7％和17.9％，CR+CRi率分别为66.4％和28.3％。然而，仍有部分存在FLT3、IDH、TP53突变及复发患者出现耐药导致疗效欠佳，FLT3下游通路PI3K/AKT、JAK/STAT5信号通路活化导致Mcl-1磷酸化及NF-κB活化，从而促使Mcl-1转录水平提高，影响疗效。有研究表明FLT3抑制剂与VEN联合应用，能够下调Mcl蛋白水平并抑制Bcl-2及Mcl与Bim结合，协同诱导凋亡，同时VEN联合FLT3抑制剂，可提高FLT3抑制剂作用[16]–[17]。因此，基于FLT3-ITD突变阳性AML原始细胞比例高、CR率低、发病初期年龄较大、化疗耐受性差及复发后再诱导疗效差等特点，结合FLT3-ITD抑制剂联合VEN+AZA方案应用可能机制，我们尝试采用索拉非尼联合VEN、AZA方案诱导治疗12例FLT3-ITD突变AML患者，其中初治8例，复发难治4例，诱导治疗后7例达到CR，3例达CRi，2例达PR，8例治疗前存在肺部、肠道感染，心功能不全患者均顺利完成诱导治疗，提示该方案具有良好的安全性及疗效，并且治疗期间新发感染率较常规化疗低，但仍需关注合并用药对VEN及索拉非尼量效的影响，尽可能治疗期间监测VEN浓度及骨髓抑制情况，适时调整用药剂量，且需要注意的是当与伏立康唑等CYP3A抑制剂同时应用时，应减低VEN浓度50％。同时，个别年轻复发患者虽然采用此方案再次获得CR，但仍需重视再次复发的问题，尽早在缓解期行allo-HSCT。综上所述，索拉非尼联合VEN+AZA方案对于老年、化疗耐受性差、复发及伴随感染患者安全、有效，且不良反应相对少，但由于随访时间及应用病例数有限，对于长期疗效及安全性仍需扩大病例数及长期随访。
